# Supplementary material for: Non-Thermal Plasma Application in Tumor-Bearing Mice Induces Increase of Serum HMGB1
Source: Int J Mol Sci. 2020 Jul 20;21(14):5128. doi: 10.3390/ijms21145128 (PMC7404183; doi:10.3390/ijms21145128)
Supplement: Supplementary file 1 [file ijms-21-05128-s001.pdf]

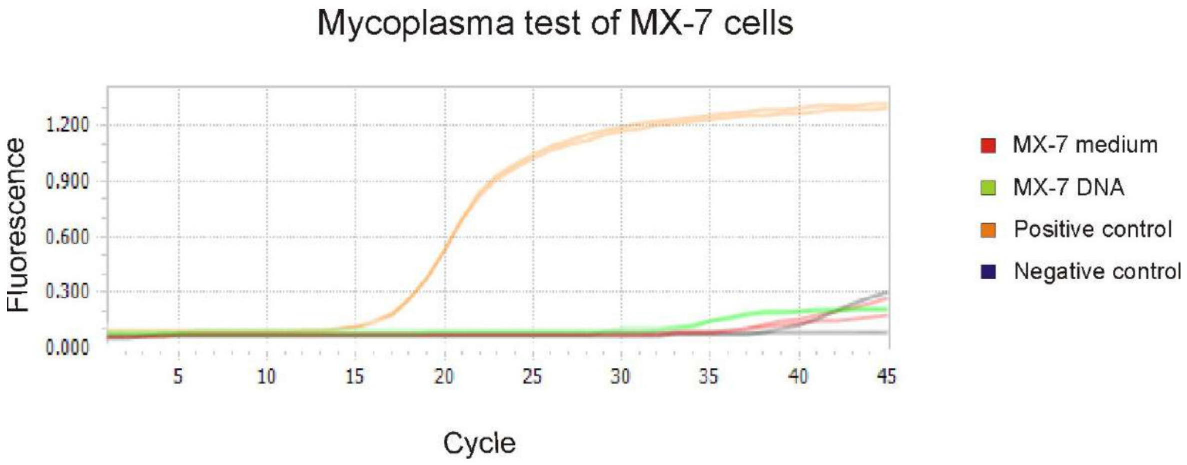

Supplementary 1. Mycoplasma test of MX-7 cells. Reverse transcription polymerase chain reaction (RT-PCR) analysis was performed on Light Cycler 96 (Roche, Roche Diagnostics International, Switzerland) and used common models and algorithms for analysis of real-time PCR data with corresponding equipment software. RT-PCT was performed in the one-tube reaction mixture BioMaster RT-PCR SYBR Blue (Biolabmix Ltd., Novosibirsk, Russia, www.biolabmix.ru) with gene-specific primers: Myc-f: 5'GGCGAATGGGTAAGTAACACG3' and 5'CGATAACGCTTGCGACCTAT3'. These primers detected various Mycoplasmas and *Ureaplasma urealyticum*.

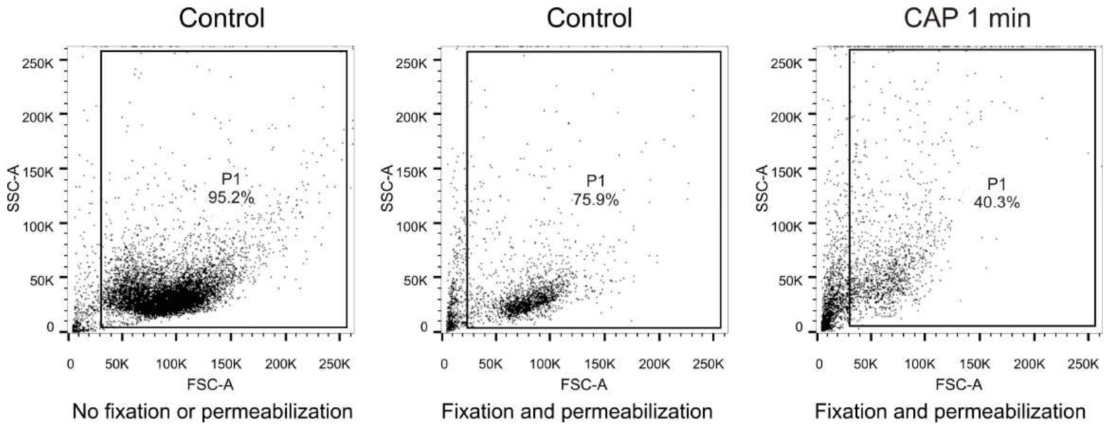

Supplementary 2. Analysis of MX-7 control and plasma-treated cells by flow cytometry in SSC/FSC graphs. These graphs were used to gate live cell population (P1) and to exclude debris.
